# Supplementary material for: Homocysteine Metabolism Pathway Is Involved in the Control of Glucose Homeostasis: A Cystathionine Beta Synthase Deficiency Study in Mouse
Source: Cells. 2022 May 25;11(11):1737. doi: 10.3390/cells11111737 (PMC9179272; doi:10.3390/cells11111737)
Supplement: Supplementary file 1 [file cells-11-01737-s001.zip › cells-1711856-supplementary.pdf]

Supplemental data

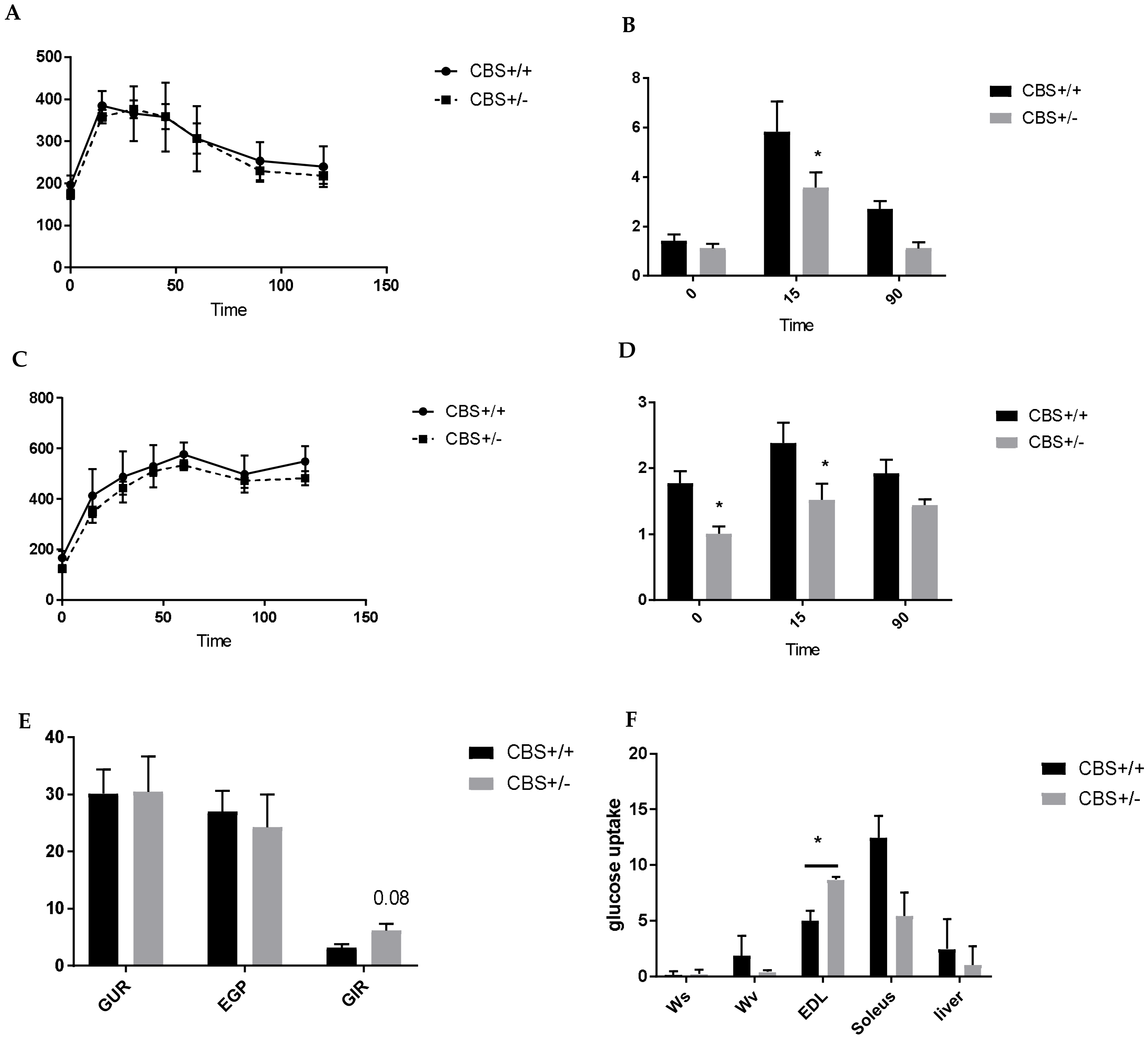

Supplemental Figure S1: Glucose homeostasis in Met-HF CBS+/- and Met-HF CBS+/+ mice. A. Oral glucose tolerance test 2g/kg bw. B. Insulin secretion at baseline and in response to OGTT. C. Intraperitoneal glucose tolerance test 2g/kg bw. D. Insulin secretion at baseline and in response to ipGTT. E. Glucose turnover rate : glucose utilization rate (GUR), Endogenous glucose production (EGP) and Glucose infusion rate during euglycemic-hyperinsulinemic clamps. F. Glucose uptake per tissue. Data are means +/- sem. * $p < 0.05$ vs. CBS +/+. Analysis by two-way ANOVA with post hoc Bonferroni test.

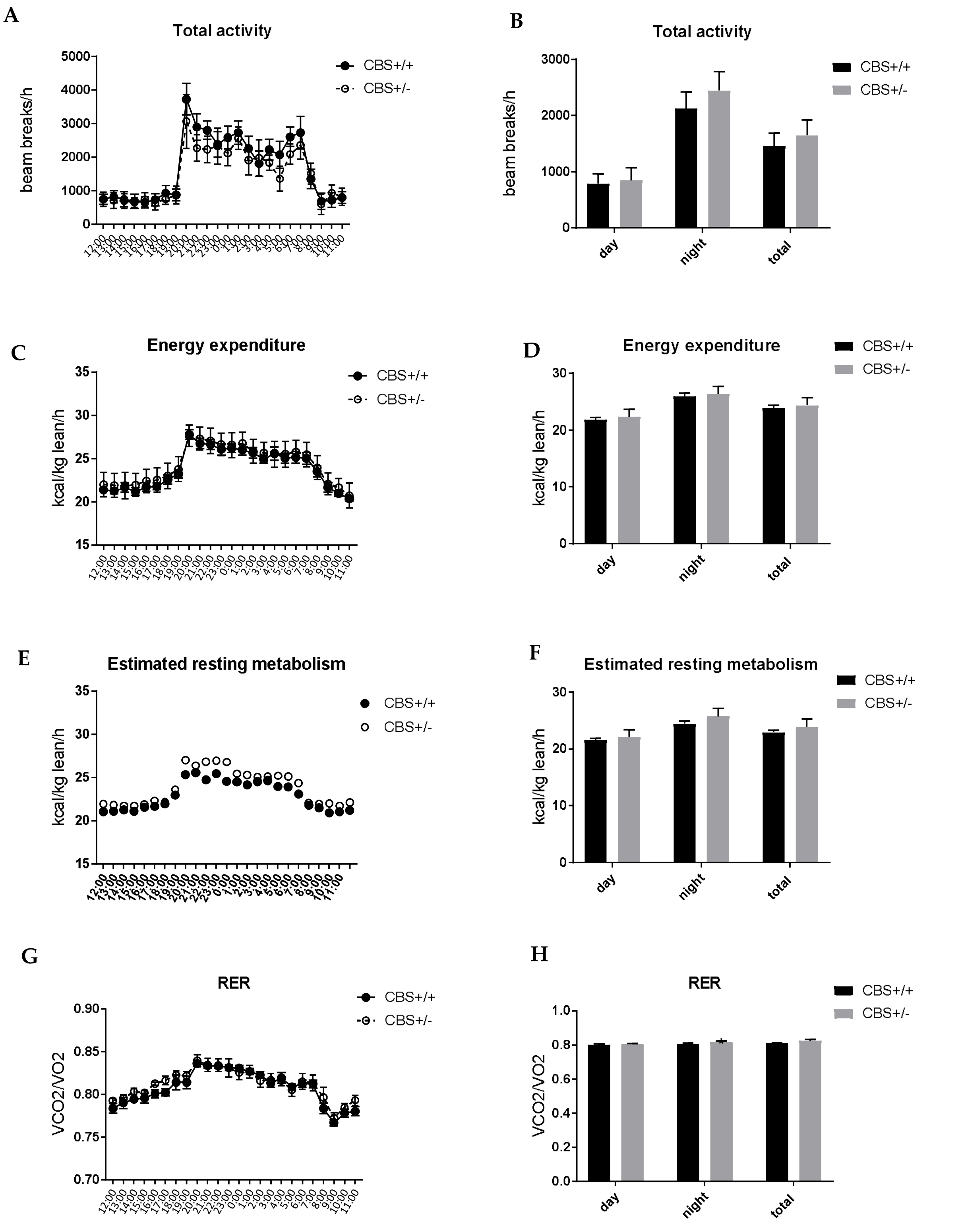

Supplemental Figure S2. Met-HF CBS+/- mice do not display metabolic change.
A, B. Total activity. C, D. Energy Expenditure. E,F. Estimated resting metabolism; G, H Respiratory Exchange Ratio (RER) of mice exposed to a high fat diet and supplemented with 0.5% methionine in drinking water. Data are represented as a representative line figure (A, C, E and G) over 24h (mean of 5 experimental days), and a bars graph showing the mean of daylight, night and 24h values (B, D, F and H). Values are expressed as mean ± sem of 5 animals minimum per group.

Supplemental Table S1
List of primers used.

Adra2a_m_S	tagaactgacttttcttccgttctc
Adra2a_m_AS	aacatacacgctcttcttcaagc
M3_m_S	ttcccatcatgatacacacca
M3_m_AS	aatgtcacgtgcttggtcac
CBS_m_S	cggatgacctgcattcatct
CBS_m_AS	gaagtggagctatcagagcat
GcK_m_S	ggccaccaagaaggaaaaggta
GcK_m_AS	cgtaggtgggcaacatctttaca
Cx36_m_S	atatccccagccagtcctct
Cx36_m_AS	cttaatgcaggcaacactgg
Glucagon_m_S	tacacctgttcgcagctcag
Glucagon_m_AS	ttgcaccagcattataagcaa
Glut2_m_S	tcttcacggctgtctctgtg
Glut2_m_AS	aatcatcccggttaggaaca
Ins1_m_S	cagagaggaggtactttggactataaa
Ins1_m_AS	gccatgttgaaacaatgacct
Ins2_m_S	gaagtggaggacccacaagt
Ins2_m_AS	agtgccaaggtctgaaggtc
Pdx1_m_S	gaaatccaccaaagctcacg
Pdx1_m_AS	cgggttccgctgtgtaag
Pax6_m_S	caccagactcacctgacacc
Pax6_m_AS	accgcccttggttaaagtc
Nkx6.1_m_S	gagttaggaggtccccaagg
Nkx6.1_m_AS	ttgctgctttcaaaccctct
Nkx2.2_m_S	tgtcccagagtcacggagt
Nkx2.2_m_AS	agggcttaagatgcctggat
Mafa_m_S	ctccagagccaggtggag
Mafa_m_AS	gtacaggtcccgctccttg
Ldha_m_S	cgccctctgctcttgattt
Ldha_m_AS	catcacccagagccctacag
Rfx6_m_S	ctgcaggaaagagaaactgga
Rfx6_m_AS	ggaaatttttggcgaattgtc
MCT1_m_S	ggatatcatctataatgttggctgtc
MCT1_m_AS	gctgccgtatttattcaccaa
Somatostatine_m_S	cccagactccgtcagtttct
Somatostatine_m_AS	gggcatcattctctgtctgg

Supplemental Table S2. Spearman correlations for 6 genes involved in homocysteine metabolism and phenotypic traits. Significant *p*-values < 0.05 are in bold and italic.

			Insulin Resistance		Stimulated Insulinemia		Basal Glycemia		Glucose Intolerance	
**Symbol**	**Module**	**Module_Membership**	**R(Spearman)**	***p*-value**	**R(Spearman)**	***p*-value**	**R(Spearman)**	***p*-value**	**R(Spearman)**	***p*-value**
Cbs	blue	0.895006292	0.748194014	***0.000355568***	0.073290835	0.552534561	-0.24822652	0.09623854	-0.031453329	0.83561374
Dyrk1a	turquoise	0.731517373	-0.663570691	***0.002677984***	0.013016758	0.916095457	0.126827467	0.4009576	0.237256776	0.11236968
Mat1a	grey	-0.048717701	-0.110423117	0.662698431	-0.025995343	0.833336483	0.41761768	***0.00388057***	-0.32711462	***0.02648781***
Mthfr	brown	0.495739946	0.1124871	0.656756563	-0.077947857	0.527504521	0.066683118	0.65971191	-0.285731907	0.05423705
Mtr	turquoise	0.784134672	-0.543859649	***0.019643564***	-0.067412299	0.58492035	-0.014434644	0.92414713	0.0853557	0.57274764
Ahcy	yellow	0.899791622	-0.73993808	***0.000447266***	-0.279383135	***0.021038604***	0.616680051	***.0000050293***	-0.313731537	***0.0337369***

			Basal Insulinemia		T15 Insulinemia		T90 Insulinemia		Plasma SMs Average	
**Symbol**	**Module**	**Module_Membership**	**R(Spearman)**	***p*-value**	**R(Spearman)**	***p*-value**	**R(Spearman)**	***p*-value**	**R(Spearman)**	***p*-value**
Cbs	blue	0.895006292	0.345268542	***0.003931356***	0.253998549	***0.036608411***	0.35041275	***0.00339506***	0.570691434	***0.01338428***
Dyrk1a	turquoise	0.731517373	-0.203343894	0.096281472	-0.110203458	0.370978568	-0.188481176	0.12373789	-0.477812178	***0.04491215***
Mat1a	grey	-0.048717701	0.325266252	***0.006799483***	0.208420812	0.088081834	0.126888391	0.3024825	-0.325077399	0.18808478
Mthfr	brown	0.495739946	0.051418101	0.677103283	-0.026567928	0.829719856	-0.146280479	0.23392404	-0.261093911	0.2953326
Mtr	turquoise	0.784134672	-0.224834905	0.065279297	-0.171584533	0.161785948	-0.137042516	0.26510868	-0.356037152	0.14702555
Ahcy	yellow	0.899791622	0.027255029	0.825384558	-0.069664465	0.572411246	-0.166226082	0.17549127	-0.364293086	0.13721672
